# Again the Question of Cancer1Part of a paper read before the Medical Society of the State of New York, at Albany, January 31, 1900.

**Published:** 1900-03

**Authors:** Roswell Park

**Affiliations:** Buffalo, N.Y.; Professor of Surgery, Medical Department University of Buffalo


### BUFFALO MEDICAL JOURNAL.

VOL. XXXIX.—LV. MARCH, 1900. No. 8.

### Original Communications.

### AGAIN THE QUESTION OF CANCER.[1]

BY ROSWELL PARK, A.M., M.D., Buffalo, N. Y.
Professor of Surgery, Medical Department University of Buffalo.

IF ONE may rely upon medical journals both at home and abroad as fair indices, the problem which both most perplexes and interests the medical world today is still that of cancer. This must be my excuse for again bringing this subject before you. No better proof of this can be given than the timely appearance of the "Cancer Number" of the *London Practitioner*, for April of last year, which aroused the greatest interest all over the world. A year ago I read before this society a paper on the same subject, in which, among other things, I dealt with figures and statistics.

To go minutely into this subject requires that kind of study of figures and of maps which can only be satisfactorily given at the desk, and it is not my purpose today to carry you into intracacies of this kind. I am perfectly willing, however, to reiterate the essential conclusion which I have repeatedly uttered and published—namely, that cancer as a disease is certainly upon the increase, and that the matter of its *rate* of increase is subsidary to this principal fact.

When voicing this opinion, as I often have to, I have been frequently asked, Is not this to be accounted for by improvement in diagnosis? And some are so sure that the answer to this should be affirmative that they dismiss the whole question with that satisfactory, self-given answer. Such reasoning is, however, fallacious. It is true that improved methods of diagnosis prevail and the disease is now recognised as formerly it was not. It is also true that certain

1. Part of a paper read before the Medical Society of the State of New York, at Albany, January 31, 1900.

lesions formerly considered cancer are not so now; actinomycosis, for instance, and other of the infectious granulomata. This, to be sure, will exclude only a certain small percentage of cases. But one feature of this matter I have never yet seen alluded to in print, and that is that just in proportion as diagnostic methods are improved and operations attempted earlier, just in that proportion do we get actual cures and save these patients from dying of the disease. If, therefore, such improvements be regarded as they should be, it will appear that mortality rates are being actually reduced as compared with the real number of cases. To me this really means a great deal, and I do not hesitate to say that it should be a matter of pride with the surgical profession that they may be able to reduce the death-rate, while, nevertheless, the disease rate is slowly creeping up. More important, then, than to study the deaths is to note the number of actual cases of cancer occurring, and I believe that the experience of every surgeon living will tally with my own in this regard.

A most cogent plea for collective investigation of cancer has been recently published by Katz, of Hamburg (*Deutche Med. Woch.*, 1899, Nos. 16, 17.) He calls attention to the unsatisfactory explanation afforded by many of the theories now in vogue, and argues logically and convincingly in favor of the method by concerted investigation, and particularly the statistical method. It was Mars d'Espine who perhaps set us our first example in the direction of statistical study, when he reported the statistics for thirteen years in the Canton of Geneva, a careful study which has not yet found a sufficient number of imitators. The special interest of Katz's paper at present for us, is the corroboration which his own studies afforded of the statement which I have so frequently made, that there is every reason to think that cancer, as a disease, is upon the increase. Katz shows, for instance, from a study of Hamburg conditions, the experience of private practice and the mortuary as well as pathological statistics of the General Hospital there, as well as by the studies of E. Fraenkel, that in that city, for example, there is a well-marked increase in the percentage of cancer. Moreover, this statement is accentuated by statistics from all over the world, which seem to show that not only is the disease upon the increase, but that the age of liability is becoming younger, and that it is rather the better classes of people than the lower in whom it seems to predominate. Katz concludes his paper by a recognition of the difficulty of collective investigation, and at the same time by a statement that it is of the greatest importance that it should be undertaken.

A topographical study of the disease will render great aid in solving some of its mysteries. As classical models for such study we may refer to Power's paper on The Local Distribution of Cancer and Cancer Houses, in the April, 1899, number of the *Practitioner*, the studies contained in the *British Medical Journal* before July 1, 1899, with the editorial comments thereon, and the paper by Lloyd Jones, on The Topographical Distribution of Cancer, in the *British Medical Journal*, for April 1, 1899.

What could be more accurate, in such work, than Jones's method, which he describes as follows:

Having obtained the list of houses in which persons had been taken ill with cancer, I proceeded to visit the houses and to mark each one on an ordnance map—scale of ten feet to a mile—showing the affected and the non-affected houses in red and black respectively. This map of the borough showed at once that certain districts were fairly free from cancer, while other regions were far more extensively affected. The areas most affected of all showed 1 case in every 2 to 5 houses; the most healthy areas, from this point of view, showed only 1 case (or no case) in every 60 houses.

The figures obtained were as follows:

Total number of houses concerned, 5,685; unaffected houses, 5,247; affected houses, 438. Showing a mean of 1 affected house to every 11.9 unaffected houses. Multiple cases in the same house divisible into: (1) Double cases, 7; (2) triple cases, 3.

But as showing what can be accomplished in rural districts in this same line of work, if only the necessary zeal and intelligence be applied to it, take the following illustration furnished me by Dr. John E. Sutton, of Albion, Orleans County. He writes me:

During the last five years, or so, there have occurred from all causes, within a range of one and one-eighth miles of a small hamlet near Albion, N.Y., (Rich's Corners) 16 deaths. Of these 9 were unquestionably from cancer; 2 were probably caused by cancer, judging from the history of the cases as learned from the friends. Of the remaining 5 one died of a pistol-shot wound, leaving 4 of the 16 as dying of disease other than cancer. There are at present 2 cases in the territory mentioned; one of them I operated upon a year ago, without recurrence as yet; the other is beyond help.

This letter is accompanied by a drawing which makes plainer yet the facts therein stated.

I wish that this report of Dr. Sutton's might stimulate many others to similar study and report.

Durante and Cohnheim believe that tumors proceed from embryonal remains; Thiersch taught a lessened resistance of connective tissue to epithelial encroachment; Hansemann and Hauser hold to an altered cell condition which they speak of as *anaplasty*, while Fabre-Domergue also believes in certain vague cell changes or cytotrophic alterations, and Ribbert regards tumor formation as unwonted cell activity and the escape of certain cell groups through proliferating connective tissue. All these statements, however, seem to be rather descriptions of certain facts observed at various times, and they still leave the real question of *why* these cells act as they do quite untouched.

It seems quite fair to assume that as a preliminary to cancer formation we must assume a certain *predisposition* of tissues, which may be general or local, inherited or acquired. This is no more than we fully believe to obtain in other diseases to which cancer shows more or less analogy. We see what local predisposition may do under conditions of chronic irritation where we have cancer following chronic eczema, catarrh, ulcers, scar formation, or gall-stone, diseased teeth, mucous patches or other syphilitic lesions, or the presence of such foreign bodies as clay pipes, soot, paraffin and the like. It is, indeed, now a question whether such chemical irritants as enzymes may not contribute to cancer formation.

Again, in the way of local predisposition we see that nearly all cases of skin cancer occur on exposed parts of the body, face, hands, genitals or nipples; and when on other parts almost invariably on surfaces where there has been some lesion as warts, eczema, sebaceous tumors, ulcers or scars. The only exception to this general statement is the hairy scalp, and yet this may be regarded as in a sense a well protected surface.

Cancer of the tongue is also found almost solely in men, because smoking, bad teeth and syphilis of the mouth mostly prevail in the male sex. Cancer of the ear begins nearly always on the upper and outer border, *i.e.*, the least resisting and most often irritated part, as by injury, frost-bite, etc., or else in those recesses which are hardest to clean. Cancer of the upper alimentary tract occurs by far most often at the sight of previous lesions or irritations, *i. e.*, scars, fissures, etc. In the stomach and bowels we know that they are often preceded by ulcers. After food has been in one sense sterilised by gastric juice, bile and pancreatic fluid, it seldom carries cancer germs, and primary cancer of the small intestine is a great rarity. In the ileo-cecal region and the colon it becomes common again, and here it is especially that stagnation of the fecal current permits it.

Particularly about the breast and nipple, where chronic mastitis, subinvolution, and ulcers or fissures furnish the local predisposition, do we meet with cancer. Here, also, inherited cell proclivities may play a large rôle. But to external influences must be assigned the major rôle, as well as to such lesions as eczema, or intertrigo of overhanging mammæ, or the pressure of corsets.

So, too, with cancer of the uterus. Chronic tissue changes furnish the site for most of these lesions, and especially scar tissue, ectropion of mucosa, etc., while cancer of the endometrium is apparently inseparable from catarrhal lesions, placental relics or diseases. Even submucous myomata are accused of giving rise to cancer.

The apparently prophylactic effect of circumcision is well known, since Jews relatively seldom get syphilis or cancer of the penis, but gonorrhea often.

Cancer of the duct glands is relatively common as compared with those of the so-called ductless glands, which latter suffer much oftener from endothelioma and angeio-sarcoma.

Summarised, carcinoma takes its origin almost always at those places where chronic tissue alterations or scars have caused local changes which we may fairly regard as constituting a predisposition, or where retention of secretion or contents have produced the same effect. While all this cannot be said for sarcoma, certain other features stand out predominantly, which are equally significant.

It is evidently manifestly impossible to draw any sharp distinction between the true tumors and numerous other infectious lesions, whose parasitic origin is not now denied. We must, moreover, never lose sight of the analogies offered from the vegetable kingdom, where practically all xylomata or tree-cancers (many of which are fatal to the trees) are due to invasion of parasites either before or after traumatism of some kind, even such as frost and ice may produce.

We must remember that sarcoma of bone, like osteomyelitis, may be apparently produced after injury or exposure, and we can then view it only as the expression of the localised action of some parasite circulating with the blood, and setting up disturbance at the point of least resistance.

Autoinfection with cancer is by no means so rare. Implantation cancer of one labium from the other is not uncommon, and the vagina is not infrequently infected from the os. When colloid, abdominal and ovarian cancers were more frequently tapped than now, implantation along the trocar tract was frequently seen. How often we see secondary cancer along the line of incision made for removal of the primary lesion, infected at the time.

Instances must be known to many of you, as they certainly are to me, of various members of the same family, in one house, succumbing one after another to cancer. I have elsewhere called attention to Behla's remarkable study of endemic cancer in the little town of Lückau.

There is no doubt but that many fungi can produce certain enzymes which have the power of exciting both cell proliferation and degeneration. So, too, Bejerinck, for example, has found that the juice pressed out from the larvæ of those insects which produce galls in trees has the same power of exciting proliferation of plant cells as have the larvæ themselves (Eckstein, Pflanzenzellen und Gallentiere, Leipzig, 1891.)

Czerny and others have recently again raised the question as to the possibility of spontaneous retrocession of malignant tumors. This is a rare phenomenon in which I thoroughly believe, and to which I propose at some later time to devote special time and study. At present I am anxious to obtain data of carefully observed cases, and solicit your aid in securing them. Believing in it, as I am compelled to do, there comes up at once the query, how many cases of improvement after employment of erysipelas toxins or after exploratory operations may be due to it alone?

(The essayist then presented a list occupying several pages of reported cases of cancer occurring at precociously early ages.)

It is never possible to get away from that question of fundamental importance—What is the cause of cancer? Realising the futility of purely personal effort in the solution of so large a problem, legislative aid was invited and finally secured. By means of this a laboratory has been well equipped and manned for the purpose. This laboratory has been organised in order to study all the problem includes, and in the broadest possible way. It is not committed to any particular view nor based upon a single idea. As the originator of that laboratory I wish to emphasise that my own papers on the subject, including this one, are not to be considered as anything more than my own personal views, save when so specified. When Dr. Gaylord thinks the time has come to publish the results of his investigations, and those of his co-laborers, then we may be said—as an institution—to stand committed to whatever views are at that time expressed. For myself, I have not hesitated to espouse the parasitic theory, for many years, and can only see in the labors of others, including those of my colleagues in the State Laboratory, constantly increasing confirmation of my beliefs. Yet these views are as largely the result of clinical as of pathological study.

Malignant tumors certainly constitute a morbid group which may be regarded as having strong family resemblances, and yet it is quite impossible to suppose that if of parasitic origin a single parasite should be at fault. One cannot imagine that epithelioma of the lip and sarcoma of bone marrow are necessarily due to the same parasite. Here again, as so many times, we find a strong analogy between cancer and the infectious diseases. Suppuration, for instance, we know may be produced by any one of numerous organisms. We acknowledge its parasitic explanation, while at the same time appreciating this fact. I want it to be emphasized that in all my own writings upon this subject I have never for a moment declared all forms of cancer to be due to a single parasite, but believe that there are numerous organisms which may produce this condition under favorable circumstances. If this be correct, we must first establish the general nature of the process and later try to identify the organism at fault.

When the State of New York by appropriation established a laboratory for the investigation of cancer it devolved upon those who had charge of the work to elaborate a rational plan by which the subject could be properly approached. Necessarily, the first steps in such an investigation had to be taken along conventional lines, and so extensive and yet scattered has been the work of various investigators of the subject, that it has required a considerable period of time and no small amount of effort to thoroughly collect and classify the material at hand. It was decided in the beginning that the subject must be as much as possible approached from all sides, but the limitations of our appropriation have thus far rendered it only possible to carry out an elaborate pathological and bacteriological research. From the beginning the plan has been to add to these two sides of the question a complete and full investigation along chemical lines. Aside from the fact that our present quarters and appropriation have thus far not permitted our undertaking this third branch, the proper man was not apparent, and it is only within a few months that such a scientist has been obtainable. In making to you a short report of the amount of work which has been accomplished, I wish to particularly emphasize that much of the work which may at first glance not appear of great importance has been none the less necessary, and has required as great care and precision as work which we are at present conducting and which gives promise of much that is positive. We may say in reviewing this portion of the work that our early efforts showed us that, while all of the various appearances described could be found in certain specimens, no method gave us constant results. Likewise, we have been able to determine to our own satisfaction that histological investigation alone will never determine the nature of certain suspicious bodies which are to be found in carcinoma. As I stated before this meeting a year ago, examination of fresh tumors gave us more encouraging results, and we were able to determine the presence in all carcinomata of certain bodies resembling fat, but which could not be dissolved in ether or other fat solvents. Various attempts to harden and stain these bodies had met with only partial success when we came into possession of an article, published by H. G. Plimmer, in the Cancer number of the *Practitioner*. Plimmer has investigated during a period of six years over 1,100 carcinomata, and by a special staining method which in principal consists in using a fat fixative, osmic acid, removing the same by oxidation with hydrogen peroxide, and staining with special reagents, has succeeded in demonstrating the presence in practically all carcinomata of certain characteristic bodies which we are now able to say are identical with those which had been observed by Dr. Gaylord in the fresh state. Plimmer's method furnishes further corroboration of the exceeding close resemblance which these bodies in the fresh state bear to fat, and it is probable that the success of his methods depends upon the fixation of fat-like substances which these bodies contain. Since employing Plimmer's method, Dr. Gaylord has been able to demonstrate in all carcinomata examined the characteristic appearances described by Plimmer. In many cases these bodies are very few in number and only prolonged search will disclose them. Plimmer reports some success in the cultivation of these organisms, and although we have as yet been unable to obtain the organisms by culture, we are at present working upon an indirect method by which we hope we shall ultimately succeed in isolating the organisms in all cases where present.

Our most recent work, by which we are introducing tumors into animals, employing another method, has resulted in the discovery of the fact that by the employment of proper staining methods the parasites found in cancer may be detected in large quantity in the enlarged lymph nodes of the experimental animal. This work is still in progress, and we are not prepared yet to publish it in full; but I wish merely to indicate to you the fact that even slight variation in technique may give totally negative or exceedingly positive results. In one case Dr. Gaylord succeeded in producing a true adenocarcinoma in an animal, by inoculation with fluid from the peritoneal cavity of a man suffering from colloid carcinoma of the omentum.

In this fluid was observed the presence of an organism which apparently belongs in the yeast group, and which we were unable to cultivate. The organism in the original fluid was injected into the jugular vein of a guinea-pig and after three weeks and a half the animal was killed; whereupon, minute nodes of beginning adenocarcinoma of the lung were found. This is the first positive case of this kind, and holds out to us the definite hope that when we are able to understand the nature of these organisms we may be able to produce carcinoma in animals with organisms obtained from carcinoma in man. The laboratory is in possession of pathogenic yeasts which various investigators, Sanfelice, Plimmer and others, have isolated from cancer, and we are now carrying on an elaborate investigation in the first steps of which we have been able to confirm all that Plimmer has published. The principal difficulty which we encounter at present is an entire lack of required knowledge, on the part even of botanists, of the nature of the organisms in question. These organisms are exceedingly polymorphic, and it requires prolonged experimentation to determine the relation between certain definite bodies found in the tissue, which are presumably these organisms, and the appearance of the same organisms when grown upon artificial culture media. In this direction our recent experiments are rendering us great aid, and we have been able to confirm the identity of the bodies described by Plimmer and observed by ourselves in the fresh state, with the organisms which Plimmer has been able to cultivate. In his examination of 1,100 tumors Plimmer has been able only in five cases to obtain a culture of the organism upon artificial culture media, indicating that the cultivatability of the organism is exceedingly variable. Therefore, the large number of cases in which we have obtained negative results indicates nothing more than the fruitlessness of attempting to study organisms of unknown habitat by conventional methods. There are certain indications that the various forms of tumor are not produced by one organism, or even necessarily by one class of organisms. This fact I brought prominently before you a year ago and today I am able to give you definite confirmation of the same, in that from one pigmented tumor we have obtained a pure culture of an unknown fungus, which under certain conditions produces a pigment identical in appearance with the pigment found in the tumor. We have likewise been able by special methods to stain the elements of the organism in the tumor. The experimental portion of our work we are not yet prepared to report upon.

In this work we have endeavored—and have succeeded to a limited extent—to arouse the interest and coöperation of the veterinarians who come into contact with a large amount of interesting material. To the personal interest of Drs. Wende and Zinc, of Buffalo, for instance, we are indebted for some very interesting specimens from animals. Such as these we are always anxious to secure, and consequently, I would appeal through you to them, so far as you have it in your power to spread this request for coöperation.

510 Delaware Avenue.

